# Using Administrative Data to Ascertain True Cases of Muscular Dystrophy: Rare Disease Surveillance

**DOI:** 10.2196/publichealth.6720

**Published:** 2017-01-12

**Authors:** Michael G Smith, Julie Royer, Joshua R Mann, Suzanne McDermott

**Affiliations:** ^1^ Bureau of Maternal and Child Health Division of Research and Planning South Carolina Department of Health and Environmental Control Columbia, SC United States; ^2^ South Carolina Budget and Control Board Revenue and Fiscal Affairs Office Columbia, SC United States; ^3^ Department of Preventive Medicine University of Mississippi Medical Center Jackson, MS United States; ^4^ Department of Epidemiology and Biostatistics University of South Carolina Columbia, SC United States

**Keywords:** muscular dystrophy, algorithm, administrative records

## Abstract

**Background:**

Administrative records from insurance and hospital discharge data sources are important public health tools to conduct passive surveillance of disease in populations. Identifying rare but catastrophic conditions is a challenge since approaches for maximizing valid case detection are not firmly established.

**Objective:**

The purpose of our study was to explore a number of algorithms in which International Classification of Diseases, Ninth Revision, Clinical Modification (ICD-9-CM) codes and other administrative variables could be used to identify cases of muscular dystrophy (MD).

**Methods:**

We used active surveillance to identify possible cases of MD in medical practices in neurology, genetics, and orthopedics in 5 urban South Carolina counties and to identify the cases that had diagnostic support (ie, true cases). We then developed an algorithm to identify cases based on a combination of ICD-9-CM codes and administrative variables from a public (Medicaid) and private insurer claims-based system and a statewide hospital discharge dataset (passive surveillance). Cases of all types of MD and those with Duchenne or Becker MD (DBMD) that were common to both surveillance systems were examined to identify the most specific administrative variables for ascertainment of true cases.

**Results:**

Passive statewide surveillance identified 3235 possible cases with MD in the state, and active surveillance identified 2057 possible cases in 5 actively surveilled counties that included 2 large metropolitan areas where many people seek medical care. There were 537 common cases found in both the active and passive systems, and 260 (48.4%) were confirmed by active surveillance to be true cases. Of the 260 confirmed cases, 70 (26.9%) were recorded as DBMD.

**Conclusions:**

Accuracy of finding a true case in a passive surveillance system was improved substantially when specific diagnosis codes, number of times a code was used, age of the patient, and specialty provider variables were used.

## Introduction

Administrative records that include insurance claims, hospital discharge datasets, and vital records have become important public health tools to understand prevalence of disease in populations [1-4]. Some studies have explored algorithms that can identify incident cases [5,6], while others used algorithms to identify prevalent conditions [2,7]. The special case of identifying rare but catastrophic conditions has emerged as a challenge since understanding the effects of these conditions on populations is important for medical, public health, insurance, and advocacy groups [8-10]. Active approaches to case finding can be effective in identifying and describing cases [11] but are time intensive and expensive. With the growing availability of administrative data sources for researchers and public health practitioners, prospects of conducting surveillance more efficiently using such data sources are intriguing. However, approaches for maximizing the validity of case detection using such data sources are not yet established.

Muscular dystrophy (MD) is a particularly challenging condition for surveillance because there are 9 types with different presentations and all types are rare. There are 2 relatively common types, Becker MD and Duchenne MD (DMD), which have childhood or young adulthood onset and are more common in males. DMD is characterized by onset of symptoms by age 4 years, followed by substantial muscle weakness in childhood and progression to loss of mobility by adolescence and high risk of mortality from respiratory and cardiac failure in young adulthood. Becker MD typically is associated with an older age of onset and slower progression of muscle weakness than DMD [12]. The prevalence of Duchenne/Becker MD (DBMD) in US males aged 5 through 24 years old, using active surveillance, is estimated to be 1.38 per 10,000 [11]. The International Classification of Diseases, Ninth Revision, Clinical Modification (ICD-9-CM) code for identification of DBMD is 359.1. However, this code includes other hereditary progressive muscular dystrophies (eg, limb-girdle), so the prevalence of DBMD cannot be isolated when using passive surveillance methods. Similarly, the International Classification of Diseases, Tenth Revision, Clinical Modification (ICD-10-CM) code used to identify DBMD, G71.0, includes other hereditary progressive muscular dystrophies.

This study was designed to explore the value added using a number of algorithms to identify cases that had diagnostic support for MD (henceforth referred to as true cases) from administrative data sources, including insurance claims and hospital discharge uniform billing datasets. The feasibility of distinguishing between DBMD and other muscular dystrophies was also investigated.

## Methods

### Overview

The 2 methods of data collection used for this project are (1) a passive surveillance system whereby data about cases of MD were ascertained through the linkage of a private and public (Medicaid) insurance program and an all-payer hospital discharge data system and (2) an active surveillance system whereby data about cases of MD were abstracted directly from medical records from medical practices that serve individuals with this condition. The passive system included all 46 counties in South Carolina, and the active surveillance was conducted in 5 target counties (combined population 1.4 million), which included 2 metropolitan centers with large university- affiliated hospital systems. The active and passive systems were independently conducted.

The active system’s data collection was completed by the Maternal and Child Health Bureau at the South Carolina Department of Health and Environmental Control (DHEC), and the passive system’s data analysis was completed by the Health and Demographics Section of the South Carolina Revenue and Fiscal Affairs (RFA) Office. DHEC is the state health department, and RFA serves as a central repository for health and human service data in South Carolina. Data usage approvals for the passive system were obtained from participating organizations from which the data originated and the South Carolina Data Oversight Council. Active data collection was conducted in accordance with established ethical principles and approved by the DHEC Institutional Review Board. Upon completion of the 2 systems, the datasets were linked at RFA. Analyses were then performed at RFA, and aggregate results were provided to investigators. 

### Passive Surveillance

The passive surveillance system relied on identification of ICD-9-CM codes from insurers and hospital discharge data. People with MD do not necessarily receive care in their county of residence, so the entire state was included in the passive system. We used ICD-9-CM codes 359.0 (congenital hereditary muscular dystrophy), 359.1 (hereditary progressive muscular dystrophy), and 359.21 (myotonic muscular dystrophy) to flag cases from administrative health databases from 1998-2012 in the passive system. The linked insurance and hospital discharge data included the following variables: MD ICD-9-CM codes, sex (male, female), age (18 years and younger, over 18 years), race (white, African American, other), other neurologic code (an ICD-9-CM code for diseases of the nervous system other than MD), setting of care (inpatient, outpatient, clinic), specialty of provider noted on the claim (neurology, cardiology, genetics, orthopedics, other), and prescription for corticosteroid (yes, no). The current standard of care for DBMD includes the prescription of steroid medication; thus, this information was included to test its utility in identifying DBMD cases.

### Active Surveillance

The active system relied on record reviews in specialty physician offices in the 5 selected counties that are served by 2 large medical centers. Medical practices for physicians in neurology, genetics, and orthopedics were identified through state licensure data and a nurse from the health department with public health surveillance authority scheduled a visit to these practices to abstract medical records with an MD ICD-9-CM code. The nurse was given 2067 records, in total, with an ICD-9-CM code for MD, without consideration of the year of service. Of these 2067 records, 384 (18.58%) were confirmed as true cases after medical record review. It should be also noted that that 1530 (74.02%) of the actively reviewed records were not in the passive system and that 124 of those individuals were determined to be true cases. Likely reasons for being omitted from the passive system were that these individuals were insured by Medicare or by a private insurer that was not in the passive system and they did not have a hospitalization within the state during the study period. The nurse abstracted information from medical records to determine instances of true cases, where there was positive clinical or genetic diagnostic support for an MD diagnosis. Active unconfirmed cases included those that had a negative clinical or genetic test result for MD and/or a diagnosis of another condition (not MD).

### Cases of MD

The 537 cases that were found in both systems were used to investigate whether passively collected variables, in addition to ICD-9-CM codes, could aid in the detection of true cases of MD without active surveillance.

### Statistical Approach

Logistic regression models and knowledge of coding practices and disease course were used to determine which passively collected variables could be useful for predicting which individuals identified in the administrative data would be confirmed as true cases by active surveillance. Models were estimated only for the subset of individuals who were identified as potential cases in both the active and passive surveillance approaches. We report coefficients and *P* values for the variables instead of odds ratios because we are using logistic regression to predict MD status and not to examine the association of this status with individual variables or to report the relative odds of having confirmed cases of MD. We considered a *P* value less than or equal to .05 to be statistically significant. Variables selected for the algorithms included (1) provider specialty (neurology, cardiology, orthopedics, genetics, or other), (2) location of service (inpatient, outpatient, or clinic), (3) number of times a code was identified on claims during the study period, (4) other neurological and muscular disease codes carried forward after an initial MD code was registered, (5) age at first coded claim, (8) sex, and (9) race. The accuracy rate was defined as the number of true cases divided by the total number of cases (true positives / true positives + false positives) and was used to assess the value of the algorithms. Textbox 1 displays the MD ICD-9-CM codes and types of MD associated with each code. First, we noted if 1 of the 3 MD codes identified any type of MD. Then, we determined if a code identified the correct type of MD. Finally, we determined how accurately code 359.1 identified cases of DBMD.

**ICD-9-CM codes for muscular dystrophy and types of muscular dystrophy associated with codes.**

359.0 Congenital hereditary muscular dystrophy:

- Benign congenital myopathy
- Central core disease
- Centronuclear myopathy
- Myotubular myopathy
- Nemaline body disease

359.1 Hereditary progressive muscular dystrophy:

- Becker
- Distal
- Duchenne
- Erb’s
- Fascioscapulohumeral
- Gower’s
- Landouzy-Dejerine
- Limb-girdle
- Ocular
- Oculopharyngeal

359.21 Myotonic muscular dystrophy:

- Dystrophia myotonica
- Myotonia atrophica
- Myotonic dystrophy
- Proximal myotonic myopathy
- Steinert’s disease

## Results

Overall, there were 2698 potential MD cases identified through the passive data system only, 1530 potential MD cases through the active data system only, and 537 potential cases identified by both the active and passive data systems. Among these, 260 were determined to be true cases of which 70 were diagnosed as DBMD.

Table 1 displays results from logistic regression models of the cases identified through both active and passive surveillance, stratified by whether the first MD code was identified on or before 18 years of age. To predict the true cases for those under age 18 years, the variables that were statistically significant were number of times the MD code was recorded during the study period, having MD identified by an inpatient claim or at least 2 outpatient claims 30 days apart, and being male. Having a visit with a specialist other than a neurologist during the study interval was the only other marginally significant predictor (*P*=.054). To predict the true cases for those identified after age 18 years, we had the following statistically significant predictors: number of times the MD case was recorded during the study period, having MD identified by an inpatient claim or at least 2 outpatient claims 30 days apart, being white, and having another neurologic syndrome coded after the first code of MD in the record. For those over 18 years, being male was only marginally significant (*P*=.054).

**Table 1:** Determining variables important in muscular dystrophy algorithm development using data from 5 South Carolina counties, 1998-2012.

		Age group ≤18 years		Age group >18 years
Parameter	Referent group	Estimate/coefficient	*P* value	Estimate/coefficient	*P* value
Number of MD^a^ codes recorded	Number of encounters with MD codes—continuous variable	0.0065	.014	0.0211	.035
MD identified by inpatient claim or 2 outpatient claims at least 30 days apart	MD code used for only 1 outpatient claim or 2 claims less than 30 days apart	1.3687	.001	1.0676	.001
Sex (male)	Female	1.1856	.001	0.5080	.054
Race (African American)	White	−0.4621	.242	−0.8774	.004
Race (other)	White	−0.7188	.120	−2.3076	<.001
Neurologist coded MD	No neurology specialist coded MD	−0.4945	.203	−0.0871	.796
Other specialist coded MD	No other specialty physician coded MD	0.6661	.054	−0.2619	.368
Other neurological syndrome coded after MD	No other neurology syndrome coded	0.1290	.838	−1.1373	.004
Patient age	In years—continuous variable	0.0449	.120	0.0158	.081

^a^MD: muscular dystrophy.

See Multimedia Appendix 1 for the number of MD cases identified in both active and passive data systems by confirmation status along with the percentage of true cases for a variety of variable combinations from the passive data. Overall, 537 cases were identified in both the passive and active systems. It should be noted that, of the 260 actively confirmed true cases, about 25% were of unknown MD type. Passively collected data with at least one 1 of any of the MD codes (359.0, 359.1, or 359.21) did not accurately predict true MD cases in general (accuracy rate 260/537, 48.4%) or true DBMD in particular (accuracy rate 136/537, 25.3%). However, 1 of the codes (359.21 for myotonic MD) did have a high probability for accurately predicting any MD (accuracy rate 88%) and for predicting myotonic MD in particular (accuracy rate 46/58, 79.3%). Restricting to data with at least 1 inpatient hospitalization code or 2 other medical claim codes marginally improved the accuracy for any MD code collected passively (accuracy rate 224/378, 59.3%, for any confirmed MD; accuracy rate 118/378, 31.2%, for confirmed DBMD). When restricting to only cases coded with the 359.1 (hereditary progressive MD) ICD-9-CM code, which is the most appropriate code for DBMD, and males less than 18 years of age at first recorded 359.1 code and 1 inpatient code or at least 2 outpatient codes, a diagnosis of a case of any type of MD was a true case 82.8% (77/93) of the time, and a diagnosis of DBMD was a true case 63.8% (51/80) of the time. If a neurologist or other specialist coded 359.1, this was indicative of a true case of any MD type 83.1% (49/59) and 82.8% (77/93) of the time, respectively, and was indicative of a true case of DBMD 66% (33/50) of the time for neurology claims and 64% (51/80) of time for other specialist. If a prescription for prednisone or prednisolone was recorded in the claims system, this was indicative of a true case of any MD type 80% of the time and was indicative of a true case of DBMD 62.9% (22/35) of the time. As more visits with the 359.1 code were identified, accuracy increased for any MD type from 78.1% (82/105) for 1 visit to 86.2% (75/87) for 3 or more visits and to 96% for 10 or more visits. Furthermore, accuracy increased for identifying a true case of DBMD from 60.0% (54/90) for 1 visit to 66.2% (49/74) for 3 or more visits and to 82% for 10 or more visits.

## Discussion

### Principal Findings

This study demonstrates the potential to improve the validity of case identification for MD in administrative (billing) data with simple measures. We found that while accuracy of linked administrative data was low when using a straightforward criterion of a single diagnosis with MD, it improved substantially when additional factors were included in the algorithm. Consideration of specific diagnosis codes and number of diagnoses present in the data appeared to have the greatest impact. The diagnosis code for congenital hereditary MD (359.0) was consistently less predictive than codes for hereditary progressive MD (359.1) and myotonic MD (359.21). Based on these findings, health services researchers need to be well versed about the limitations of using ICD-9-CM codes; for rare conditions, they need to be confident that the population from which the study group is identified is large enough to produce meaningful results. Accuracy increased substantially with the number of times a diagnosis of MD occurred, with the bulk of the improvement occurring between 1 and 8 diagnoses. These results were similar to a study by Kaye et al [13], which found that using (1) the specific code for amyotrophic lateral sclerosis (ALS) versus other motor neuron disease ICD-9-CM codes, (2) the code for ALS recorded on multiple visits, and (3) the ALS code from a neurology specialty claim all increased the ability to identify true cases of ALS from administrative data. The similarity of these algorithms is encouraging in that it suggests that this process may be generalizable to other rare neurological conditions.

### Limitations

This research has a number of important limitations. First, we only included data from South Carolina, a state with a population of 4.8 million residents in the southeastern United States. Replication in other geographic locations would be helpful for assuring generalizability. Second, the study was conducted using ICD-9-CM codes, and as of October 1, 2015, health providers have converted to ICD-10-CM codes; thus, our study provides insight into the identification of cases prior to 2015. It may not be appropriate to extrapolate the findings of this study to research using ICD-10-CM data. However, research on rare conditions using administrative data will continue to rely on ICD-9-CM coding for some time, given the limited sample sizes that will be available in ICD-10-CM coded records for several years.

Third, the cases in the passive system were identified if the individual received a service during the period 1998-2012, but the active surveillance was not limited by service date. This probably contributed to a number of cases that were identified by the active system. Fourth, the study would have been improved if we could have done active surveillance throughout the state, but this was not feasible due to financial limitations. We believe there were cases found in the passive system that were not identified through active surveillance because they received care in other counties. Finally, small cell sizes impaired our ability to conduct some analyses, particularly for myotonic MD. Additional research using data from multiple geographic regions may be necessary to establish the validity of billing data to identify individuals with myotonic MD.

The passive system included all provider specialty types, other professional claims, and coded facility claims from all counties within the state whereas the active system only included selected specialty practices in 5 counties. Therefore, it was anticipated that a number of cases would be present in the passive system but not found in the active one. Likewise, the active system identified cases from some payer sources that were not available in the passive system.

There are important potential advantages to using administrative data to study health care utilization and health outcomes for individuals with MD. First, the low prevalence of MD means that identifying affected individuals for enrollment in primary research studies can be very time consuming and expensive. Second, the range of data available from billing records is excellent for answering research questions related to receipt of services, number and causes of emergency department visits and hospitalizations, and health care expenditures. In some states, including South Carolina, linkable data warehouses exist, facilitating linkage to other data sources such as vital records, which enables research investigating risk of death and specific causes of mortality. Another benefit of research using secondary data is that it is not subject to limitations in recall on the part of study participants, family members, or health care providers since billing is conducted prospectively at the time of care delivery.

In applying algorithms to improve accuracy of billing data for identifying cases of MD, it is important to keep in mind the ultimate goals of the research. If the goal is to identify potential demand for resources, it may be preferable to maximize sensitivity to avoid insufficient resource allocation. On the other hand, if the goal is to evaluate the receipt and potential benefits of specific health care services for individuals with MD, using algorithms to maximize specificity and accuracy is likely to be preferable. For other types of research questions, it may be that conducting sensitivity analyses over a range of assumptions is the best approach. In every case, it is important to keep in mind the balance of sensitivity and specificity, as increasing one reduces the other.

### Conclusion

Administrative records have become important public health tools to understand prevalence of disease in populations. We explored the identification of a rare but catastrophic condition, muscular dystrophy, to maximize the validity of case detection using such data sources. Accuracy was low when using a straightforward criterion of a single code for MD; however, it improved substantially when additional administrative variables were included in the algorithm. Consideration of specific diagnosis codes, number of times a code was used, and demographic variables appeared to have the greatest impact on accuracy.

## Appendix

Multimedia Appendix 1 Variables for algorithm development from the passive data system to identify cases of muscular dystrophy in 5 South Carolina counties, 1998-2012.

## Abbreviations

ALS: amyotrophic lateral sclerosis
DBMD: Duchenne/Becker muscular dystrophy
DHEC: Department of Health and Environmental Control
DMD: Duchenne muscular dystrophy
ICD-9-CM: International Classification of Diseases, Ninth Revision, Clinical Modification
ICD-10-CM: International Classification of Diseases, Tenth Revision, Clinical Modification
MD: muscular dystrophy
RFA: Revenue and Fiscal Affairs
