# Supplementary material for: Using Administrative Data to Ascertain True Cases of Muscular Dystrophy: Rare Disease Surveillance
Source: JMIR Public Health Surveill. 2017 Jan 12;3(1):e2. doi: 10.2196/publichealth.6720 (PMC5269556; doi:10.2196/publichealth.6720)
Supplement: Multimedia Appendix 1 [file publichealth_v3i1e2_app1.pdf]

Multimedia Appendix 1. Variables for algorithm development from the passive data system to identify cases of muscular dystrophy, in five SC counties, 1998-2012									
	Any **MD***			***Correct*** **MD type****			***DBMD****		
**Passive Data System Element**	**Active Confirmed**	**Active Not-Confirmed**	**Accuracy**	**Active Confirmed**	**Active Not-Confirmed**	**Accuracy**	**Active Confirmed**	**Active Not-Confirmed**	**Accuracy**
At Least 1 Code									
Any MD Code – 359.0, 359.1 or 359.21	260	277	48%	260	277	48%	136	401	25%
Congenital Hereditary MD – 359.0	68	146	32%	DS	DS	5%	n/a		
Hereditary Progressive MD – 359.1	175	172	50%	119	228	34%	70	277	20%
Myotonic MD – 359.21 only	DS	DS	88%	46	12	79%	n/a		
1 Inpatient or 2 Other Medical Claims[1]									
Any MD Code – 359.0, 359.1 or 359.21	224	154	59%	224	154	59%	118	260	31%
Congenital Hereditary MD – 359.0	47	73	39%	DS	DS	8%	n/a		
Hereditary Progressive MD – 359.1	149	99	60%	107	141	43%	63	185	25%
Myotonic MD – 359.21 only	DS	DS	88%	DS	DS	84%	n/a		
Males <=18 years of age with Code 359.1									
At Least 1 Code	82	23	78%	57	33	63%	54	36	60%
1 Inpatient or 2 Other Medical Claims[1]	77	16	83%	54	26	68%	51	29	64%
No 359.0 or 359.21 Code	34	13	72%	26	15	63%	26	15	63%
359.1 Coded Neurology Claim	DS	DS	83%	35	15	70%	33	17	66%
359.1 Coded Other Specialty Claim (Cardiology, Genetics, Pulmonology, Orthopedics)	77	16	83%	54	26	68%	51	29	64%
No Other Syndrome	65	17	79%	48	24	67%	45	27	63%
Pharmacy Claim for Steroids†	DS	DS	80%	22	13	68%	22	13	68%
3 or More 359.1 Codes	75	12	86%	52	22	70%	49	25	66%

5 or More 359.1 Codes	DS	DS	91%	49	14	78%	46	17	73%
7 or More 359.1 Codes	DS	DS	94%	DS	DS	82%	43	12	78%
10 or More 359.1 codes	DS	DS	96%	DS	DS	87%	DS	DS	82%

DS – data suppressed due to small numbers
†Steroids include prescriptions for the following: prednisone or prednisolone
*Counted if code identified a true case of any type of MD (actively confirmed cases with unknown type were included in this category).
**Counted if code identified the correct type of MD or DBMD. Does not include active cases classified as unknown, except when any MD Code is included in the algorithm.
